# Nasal eosinophilia and its clinical correlates in patients with allergic rhinitis at a Kenyan tertiary hospital

**DOI:** 10.3389/falgy.2025.1715848

**Published:** 2026-02-03

**Authors:** Vivyane Wamara, Catherine Irungu, Musa Kipingor, Anne Barasa

**Affiliations:** 1 ENT Unit, Department of Surgery, University of Nairobi, Nairobi, Kenya; 2 ENT Department, Kenyatta National Hospital, Nairobi, Kenya; 3 Immunology Unit, Department of Human Pathology, University of Nairobi, Nairobi, Kenya

**Keywords:** allergic rhinits, nasal eosinophilia, nasal cytology, ARIA guidelines, Kenya

## Abstract

**Introduction:**

Allergic Rhinitis (AR) is an IgE- mediated inflammatory disease of the nasal mucosa. While the diagnosis mainly relies on a typical clinical history and physical examination, the evaluation of eosinophils in nasal smears is considered a direct marker of inflammation and can play a role in the diagnosis and monitoring of treatment response. The aim of this study was to evaluate the levels of nasal eosinophils among patients with AR, and to correlate these levels with disease severity and frequency.

**Material and methods:**

This was a descriptive cross-sectional study conducted at a tertiary referral hospital in Kenya from November 2022 to January 2023. Patients were diagnosed with AR using the Score for Allergic Rhinitis (SFAR) questionnaire. A Total Nasal Symptom Score (TNSS) questionnaire was administered to evaluate for nasal symptoms and assign severity scores. The patients were categorized according to the Allergic Rhinitis and its Impact on Asthma (ARIA) guidelines into either intermittent or persistent allergic rhinitis groups, and further sub-classified into mild, or moderate-to-severe AR. Nasal smears were collected to quantify eosinophil counts.

**Results:**

The study included 73 patients comprising 35 (47.9%) males and 38 (52.1%) females. The median age of the participants was 19 years (Interquartile Range 9–38), with an age range of 5–84 years. Most patients presented with intermittent symptoms (58.9%) and moderate-to-severe disease (61.6%). Pathological levels of eosinophils were found in 15 patients (20.5%). However, there was no statistically significant correlation between the level of nasal eosinophilia and either disease severity (*p* = 0.23) or symptom frequency (*p* = 0.80).

**Conclusion:**

In this study, nasal eosinophils counts did not correlate with the severity or frequency of AR as defined by the ARIA classification. These findings suggest that while the presence of nasal eosinophilia is useful for confirming the underlying type 2 inflammation in AR, it should not be used as a standalone biomarker to determine disease severity or guide management.

## 1 Introduction

Allergic rhinitis (AR) is an IgE-mediated inflammatory disorder of the nasal mucosa that occurs in susceptible individuals upon exposure to inhaled allergens. It affects approximately 400 million people worldwide, with a prevalence of 10%–30% in adults, and over 40% in children (1). In Kenya, the prevalence of AR is estimated at approximately 13% among adults and 38.6% amongst school-going children, with seasonal peaks observed in January, July and December (2, 3). The prevalence patterns of AR vary geographically due to differences in the seasonality, type, potency, and burden of local aeroallergens, which include house dust mites, dust, animal dander and pollen from trees, grass and weeds (4). Furthermore, factors such as urbanization and environmental pollution can exacerbate airway inflammation, leading to increased sensitization and allergic reactivity (5). Although the aeroallergen profile in Kenya has not been extensively studied, house dust mites, indoor mould, dog, cockroach and grass pollen are reported as the most common sensitizing allergens (6, 7).

The characteristic clinical symptoms of AR are paroxysmal sneezing, watery rhinorrhea, nasal itching and nasal congestion (8). However, the impact of AR extends beyond these nasal symptoms, significantly reducing quality of life through frequent hospital or clinic visits, impaired school and work performance and increased socioeconomic costs. Allergic Rhinitis is categorized as either intermittent or perennial, based on the time and duration of symptom occurrence, and as mild or moderate-to-severe, according to its impact on daily activities. The disease severity partly correlates with the intensity of inflammation, and is a key factor in guiding treatment decisions (9).

The diagnosis of AR is primarily clinical, based on a detailed patient history of nasal symptoms, individual history of atopy, positive family history of allergies and characteristic examination findings such as clear rhinorrhea and pale bluish tinged nasal mucosa (10). Confirmatory laboratory tests are indicated when the diagnosis Is uncertain, response to empiric treatment is poor, or to identify specific allergens for targeted management such as allergen avoidance and immunotherapy (11, 12). These tests include total serum immunoglobulin E (IgE), skin prick tests (SPT), serum allergen-specific IgE, nasal cytology for eosinophilia and nasal provocation tests. While these tests are valuable, they are often inaccessible or prohibitively expensive in resource-limited settings.

Eosinophils are the principal effector cells in the pathology of AR, and the associated mucosal inflammation in AR is characterized by tissue eosinophilia. Following allergen exposure in sensitized individuals, eosinophil levels in nasal secretions rise within hours, and their concentration has been shown to correlate with both the clinical symptoms and immunological parameters in AR (13). Consequently, the evaluation of nasal smear eosinophils serves as an objective biological marker for the type 2 inflammation that is characteristic of AR. Multiple studies have demonstrated that individuals with AR have significantly higher nasal eosinophil counts than the general population, and that these counts correlate with disease severity (14, 15). This makes nasal cytology a simple, economical and non-invasive tool for both diagnosing AR and monitoring response to treatment.

Despite the global burden of AR, there is paucity of data from Africa on its frequency, severity, and correlation with locally accessible biomarkers. This study aimed to quantify nasal eosinophil levels in patients with a clinical diagnosis of AR at a tertiary hospital in Kenya, and to correlate these levels with disease severity and frequency.

## 2 Materials and methods

### 2.1 Study design, population and recruitment

This was a descriptive cross-sectional study that recruited patients treated at the Ear, Nose, and Throat (ENT) department of Kenyatta National Hospital in Nairobi, Kenya, between November 2022, and January 2023. The diagnosis of allergic rhinitis was based on clinical assessment, including a detailed history and physical examination, and confirmed using the Score for Allergic Rhinitis (SFAR) questionnaire. The SFAR questionnaire is a validated structured allergy questionnaire with high sensitivity and specificity for diagnosing AR, making it suitable for use in resource-limited settings (16, 17). Patients with an SFAR score of ≥ 7 were enrolled into the study.

The exclusion criteria were conditions or medications known to modify eosinophil levels or disease severity, including pregnancy, sinusoidal malignancy, acute respiratory infections, nasal surgery within the previous six months, use of intranasal steroids within the last month, oral steroids within the previous six weeks, or antihistamines within the previous one week. The study population did not include patients with confirmed chronic rhinosinusitis or nasal polyposis.

### 2.2 Data collection and clinical assessment

Demographic information and clinical data related to the participants' illness and severity of symptoms were collected using the SFAR and the Total Nasal Symptom Score (TNSS) questionnaire. The TNSS, a widely validated tool, was used to evaluate the severity of four key nasal symptoms: obstruction, itching, sneezing and rhinorrhea (18). Each symptom was assigned a score from 0 to 3 (0 = absent, 1 = mild, 2 = moderate and 3 = severe) and the individual scores for the four symptoms summed up to a total score that was graded as mild (score <6), moderate (score 6–8) or severe (score 9–12).

In line with the Allergic Rhinitis and its Impact on Asthma (ARIA) guidelines, participants were categorized into either intermittent allergic rhinitis (IAR) or persistent allergic rhinitis (PAR), based on the symptoms interference with daily activities, sport or leisure, work or school, and disruption of sleep (19). They were further sub-categorized into mild and moderate-to-severe groups.

### 2.3 Nasal smear collection and eosinophil evaluation

Nasal smears were collected from all participants during anterior rhinoscopy. Briefly, a sterile swab was used to scrape the mucosa of the medial surface of the inferior turbinate, and collected secretions were spread thinly onto a glass slide, which was air-dried, fixed in 95% alcohol, and stained with May-Grunwald Giemsa stain. The slides were examined under light microscopy to record the eosinophil counts. To ensure accurate and consistent results, a single pathologist, blinded to the clinical data, evaluated all the smears. After assessing for cellularity, and in areas where the cells were well spread, ten microscopic high-power fields were randomly selected and the number of cells counted; the number of eosinophils were documented as a percentage of the total cell count. The level of eosinophilia was categorized into four groups: less than 5 per cent eosinophils (score of 1); 5 to less than 10 per cent eosinophils (score of 2); 10 to less than 50 per cent eosinophils (score of 3); and 50 per cent or more eosinophils (score of 4) (Table 1). A score of 1 was considered to reflect normal findings.

**Table 1:** Scale used to interpret nasal eosinophilia.

Eosinophilia score	% Eosinophils in nasal smears	Description	Pathology
1	< 5	No eosinophilia	Normal findings
2	5 to < 10	Slight eosinophilia	Pathology doubtful
3	10 to <50	Moderate eosinophilia	Pathological
4	≥ 50	Marked eosinophilia	Pathological

### 2.4 Ethical considerations

The study was approved by the University of Nairobi/Kenyatta National Hospital Ethics and Research Committee. Written informed consent was obtained from all participants prior to their inclusion in the study.

### 2.5 Statistical analysis

Normally distributed data were analyzed using the Student's *t-*test, while non-normally distributed data were analyzed using the Mann–Whitney U test. Categorical data were analyzed using chi-square test. Spearman's correlation was used to evaluate the relationship between nasal smear eosinophil levels and disease frequency and severity. A *p*-value of less than 0.05 was considered significant.

## 3 Results

A total of 73 patients were included in the study, comprising of 35 (47.9%) males and 38 (52.1%) females. The median age of the participants was 19 years (Interquartile Range 9–38), with an age range of 5–84 years. Detailed demographic characteristics of the study population are summarized in Table 2.

**Table 2:** Demographic characteristics of patients with allergic rhinitis.

Characteristic	Frequency (*n* = 73) *n* (%)
Sex: Male	35 (47.9)
Age group (yr)	
<10	20 (27.4)
11–20	19 (26.0)
21–30	10 (13.7)
31–40	11 (15.1)
41–50	5 (6.8)
51–60	4 (5.5)
>60	4 (5.5)

The most frequently reported clinical symptoms were sneezing in 70 (95.9%) patients and rhinorrhea in 69 (94.5%) patients (Table 3). Itchy, watery eyes were the least common symptom, reported by 58 (79.5%) patients. A family history of atopic disease (asthma, eczema or allergic rhinitis) was reported by 52 (71.2%) patients. The most common trigger for AR nasal symptoms was house dust, reported by 69 (94.5%) participants. Irritants such as perfume, sprays and soaps were reported as triggers by a minority of participants.

**Table 3:** Clinical characteristics of patients with allergic rhinitis.

Characteristic	Frequency (*n* = 73) *n* (%)
Symptoms of allergic rhinitis	
Sneezing	70 (95.9)
Rhinorrhea	69 (94.5)
Nasal congestion	67 (91.8)
Itchy-watery eyes	58 (79.5)
Triggers of allergic rhinitis	
House dust	69 (94.5)
House dust mites	16 (21.9)
Pollen	23 (31.5)
Animals	21 (28.8)
Cold	16 (21.9)
Smoke	6 (8.2)
Cigarettes	2 (2.7)
Perfume	10 (13.7)
Spray	1 (1.4)
Soap	3 (4.1)

According to the ARIA guidelines, 45 (61.6%) patients were classified with moderate-to-severe disease, while 28 (38.4%) had mild disease. Regarding symptom duration, 43 (58.9%) had intermittent AR while 30 (41.1%) had persistent AR. Significant nasal eosinophilia (a score of ≥3) was detected in 15 (20.5%) patients. Of these, 12 patients had moderate-to-severe disease, while 3 had mild disease. However, there was no statistically significant correlation between the level of nasal eosinophilia and disease severity (Rho = 0.14, *p* = 0.23), as detailed in Table 4.

**Table 4:** Nasal eosinophilia level by disease severity.

Nasal eosinophilia score	Mild disease *n* (%)	Moderate-to-Severe disease *n* (%)	*p*
1	22 (30.1)	30 (41.1%)	Rho = 0.14
2	3 (4.1)	3 (4.1)	*p* = 0.23
3	1 (1.4)	7 (9.6)	
4	2 (2.7)	5 (6.8)	
Total	28 (38.4)	45 (61.6)	

Of the 15 patients with pathological nasal eosinophilia, 9 were in the intermittent AR group while 6 had persistent AR (Table 5). However, there was no significant correlation between nasal smear eosinophilia and the frequency of AR symptoms (Rho = 0.029, *p* = 0.80).

**Table 5:** Nasal eosinophilia level by symptom duration.

Nasal eosinophilia score	Intermittent allergic rhinitis *n* (%)	Persistent allergic rhinitis *n* (%)	*p*
1	31 (42.5)	21 (28.8)	Rho = 0.029
2	3 (4.1)	3 (4.1)	*p* = 0.80
3	6 (8.2)	2 (2.7)	
4	3 (4.1)	4 (5.5)	
Total	43 (58.9)	30 (41.1)	

## 4 Discussion

Allergic rhinitis is a common allergic disease that causes significant morbidity among children and adults worldwide. It manifests before the age of 20 years in 80% of the cases (1). Our study's demographic findings are consistent with this, as the median (IQR) age of our patients was 19 (9, 38) years, with a peak prevalence in the first and second decades of life.

The diagnosis and follow-up of patients with AR in our setting is hampered by the limited access to specialized tests, such as skin prick tests and nasal provocation tests. The use of the SFAR questionnaire in this study strengthened the reliability of our clinical diagnosis of AR. The main objective of this study was to evaluate the levels of nasal eosinophils among patients with AR, and to correlate the nasal eosinophil levels with the clinical severity and frequency of AR in Kenyan patients. Nasal eosinophil counts have long been considered as a useful biomarker for AR (20).

Our primary finding was that pathological nasal eosinophils was present in 20.5% of patients; most patients had intermittent disease (58.9%), contrasting with other studies that report persistent RA as typically more prevalent than intermittent AR (21–23), and additionally, majority of the patients (61.6%) had moderate-to-severe disease, highlighting the significant morbidity associated with AR, regardless of its frequency (24). The eosinophil levels however did not statistically correlate with disease severity or frequency, as per the ARIA classification.

These findings contrast with some studies that found significant correlation between nasal eosinophil counts severity of AR (25, 26), but align with other studies which found no correlation between nasal eosinophil counts and either total nasal symptom score, or with the frequency and severity of disease, respectively (14, 27). These discrepancies in the literature suggest that while nasal eosinophils confirms and allergic inflammatory process, its utility as a reliable standalone biomarker for disease severity is questionable.

Several factors may explain the discrepant study findings. These may be attributed to different study designs, patient selection and predominant aeroallergens in different geographical areas. In our setting, the predominant symptom trigger reported by the participants was house dust (94.5%), consistent with previously reported literature identifying house dust mites as the main aeroallergen in Kenya, responsible for allergic sensitization in 76.4% of patients (6). The warm, humid conditions in many Kenyan homes create an ideal environment for house dust mites, with constant high-level indoor exposure. This chronic indoor allergen exposure may establish a high baseline of inflammation that is less variable than that seen with seasonal allergen exposure such as pollen, potentially weakening the correlation between cell counts and symptoms severity at a given time. It is also considered that eosinophil numbers tend to be lower in those with constant as opposed to seasonal allergen exposure, and similarly to Berkiten et al., we found a lower rate of eosinophilia in persistent AR patients (15). Other studies report that when the exposure to the offending allergen is weak but persistent, such as in dust mite allergy, symptoms may be of low intensity, but a minimal persistent inflammation that is predominantly neutrophilic is present (28). This differs from seasonal allergies like those due to pollen, where inflammatory pathology and markers may more closely relate with acute allergen exposure. Pollen was the next most frequently reported symptom trigger in our study population, by 32% of the participants. A previous study has reported 34.5% pollen sensitization (6). However, the pollen profile and seasonality, and association with AR symptoms in Kenya is under-researched.

This study was conducted in an urban setting, thus the role of air pollution in the pathogenesis of AR cannot be excluded. Pollutants can initiate nasal mucosal inflammation that primes the immune system towards an allergic response, such that a person with AR in a polluted environment may experience more severe symptoms. This pollution-driven hyper-reactivity could contribute to a high ARIA severity score independent of the degree of eosinophilic inflammation, as other cells can be involved in the inflammatory pathology. This could explain the lack of correlation observed in our study.

Our cross-sectional study design evaluated the nasal eosinophilia at a single time point, which may not adequately reflect the chronic, fluctuating nature of symptoms in AR patients, based on allergen exposure, weather and exposure to irritants. Also, while eosinophils are the predominant cell type in Type 2 inflammation, other cells and mediators also contribute to symptoms, thus explaining our discrepant findings.

A limitation to our study is that we did not perform specific allergen sensitization tests, which would have allowed us to better correlate eosinophilia with sensitization to specific triggers.

## 5 Conclusion

In our study cohort, nasal eosinophils counts did not correlate with the severity or frequency of AR as defined by the ARIA classification. This suggests that while the presence of nasal eosinophilia can help confirm the underlying type 2 inflammation in AR, and thus differentiate it from non-allergic AR in resource-limited settings, it should not be used as a standalone biomarker for determining disease severity.

Future studies should focus on local aeroallergen monitoring, and correlation of disease severity and frequency with more objective environmental data such as local pollen counts and pollution levels, to better understand the complex genetic and environmental interactions that drive allergy in Africa.

## Data Availability

The raw data supporting the conclusions of this article will be made available by the authors, without undue reservation.
